# 503 Social Media as a Platform for Burn Survivor Outreach at a Safety Net Hospital

**DOI:** 10.1093/jbcr/irac012.134

**Published:** 2022-03-23

**Authors:** Rachel A Colbath, Erin E Ross, Peggy J Ebner, Jeremy Yu, Justin Gillenwater, Haig A Yenikomshian

**Affiliations:** Keck School of Medicine of USC, Carlsbad, California; Keck School of Medicine, University of Southern California, Los Angeles, California; University of Southern California, Los Angeles, California; University of Southern California, Los Angeles, California; USC/LAC+USC Medical Center, Los Angeles, California; USC/LAC+USC Medical Center, Los Angeles, California

## Abstract

**Introduction:**

Outpatient follow-up is a critical component of burn recovery. Sociodemographic variables that prevent patients from pursuing follow-up can lead vulnerable groups to have a lower quality of life after burn injury. Social media provides a platform for improvement of patient outreach and support. The purpose of this study is to investigate accessibility and interest in social media interventions among socioeconomically disadvantaged and minority burn patients.

**Methods:**

Patients receiving treatment at a burn clinic in a large public hospital were asked to complete a survey about social media usage, difficulty attending follow-up appointments, and interest in engaging with the hospital through social media. Patient demographics and clinical data were obtained via chart review. The relationship between clinical or demographic factors, and interest in social media engagement, was assessed via exact logistic regression.

**Results:**

Data were collected from 65 eligible patients. Social media use among participants (76.9%) was similar to the proportion in the U.S. general population (72%). 61.5% of participants used Facebook, 40% used Instagram, and 4.6% used Twitter. 81% of participants had consistent internet access. 58.3% of respondents expected to encounter challenges when planning follow-up appointments. Challenges included difficulty with transportation (26.2%), trouble taking time off work (9.8%), and forgetting to schedule (9.8%). Participants were asked if they were interested in receiving post-discharge education, care team outreach, and follow-up reminders via social media. 36.5% of patients were very or moderately interested, 27% were slightly interested, and 36.5% were unsure or not at all interested.

20% of patients both expected follow-up challenges and were moderately or very interested in social media engagement. While controlling for social media use, the odds of having moderate or greater interest in the post-discharge engagement program were estimated to be 2.6 times higher for patients older than 39 (OR 3.64; 95% CI 1.03-14.24; *P*=.044). There was a pattern of lower interest in social media engagement with higher %TBSA, while controlling for age or social media use, though *P* values were higher than .05.

**Conclusions:**

Over half of the burn patients surveyed expected to face challenges when planning follow-up appointments, a third of whom were moderately or very interested in social media engagement. Social media may be an alternative form of outreach with older patients in particular. Observed overlap between follow-up difficulty and outreach program interest may suggest such a program could ameliorate follow-up challenges.

